# Usefulness of *Bifidobacterium longum* BB536 in Elderly Individuals With Chronic Constipation: A Randomized Controlled Trial

**DOI:** 10.14309/ajg.0000000000002028

**Published:** 2022-10-03

**Authors:** Tsutomu Takeda, Daisuke Asaoka, Shuko Nojiri, Naotake Yanagisawa, Yuji Nishizaki, Taro Osada, Shigeo Koido, Akihito Nagahara, Noriko Katsumata, Toshitaka Odamaki, Jin-Zhong Xiao, Toshifumi Ohkusa, Nobuhiro Sato

**Affiliations:** 1 Department of Gastroenterology, Juntendo University School of Medicine, Tokyo, Japan;; 2 Department of Gastroenterology, Juntendo Tokyo Koto Geriatric Medical Center, Tokyo, Japan;; 3 Department of Medical Technology Innovation Center, Juntendo University School of Medicine, Tokyo, Japan;; 4 Department of Gastroenterology, Juntendo University Urayasu Hospital, Chiba, Japan;; 5 Department of Gastroenterology and Hepatology, The Jikei University Kashiwa Hospital, Kashiwa, Japan;; 6 Next Generation Science Institute, Morinaga Milk Industry Co., Ltd., Zama, Japan and; 7 Department of Microbiota Research, Juntendo University Graduate School of Medicine, Tokyo, Japan.

## Abstract

Few reports exist regarding the therapeutic effects of probiotics on chronic constipation in elderly individuals. This study evaluated the effects of *Bifidobacterium longum* BB536 in elderly individuals with chronic constipation.

**METHODS:**

This was a randomized, double-blind placebo-controlled, parallel-group superiority trial in Japan (UMIN 000033031). Eighty older adults diagnosed with chronic constipation were randomly assigned (1:1) to receive either probiotics (*B. longum* BB536, 5 × 10^10^ colony-forming unit, n = 39) or placebo (n = 41) once daily for up to 4 weeks. The severity of constipation was evaluated using the Constipation Scoring System. The primary end point was the difference in the changes from baseline in the constipation scoring system total score between the 2 groups at week 4.

**RESULTS:**

A total of 79 patients (mean age of 77.9 years), including 38 patients in the BB536 group and 41 in the placebo group, completed the study. The primary end point was not significant (*P* = 0.074), although there was significant improvement (*P* < 0.01) in the BB536 group from baseline to week 4, but there were no significant changes in the placebo group. There was a significant difference and a tendency toward a difference in the changes from baseline on the stool frequency (*P* = 0.008) and failure of evacuation (*P* = 0.051) subscales, respectively, at week 4 between the 2 groups. Few adverse events related to the probiotics were observed.

**DISCUSSION:**

The primary end points were not significant. However, probiotic supplementation significantly improved bowel movements. These results suggest that *B. longum* BB536 supplementation is safe and partially effective for improving chronic constipation in elderly individuals.

## Graphical abstract

**Figure:**
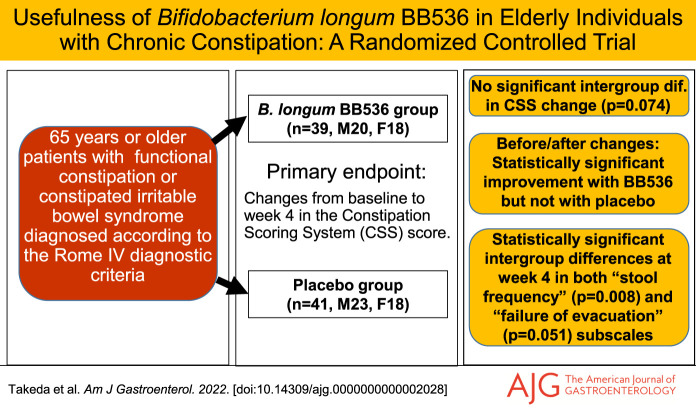

## INTRODUCTION

Chronic constipation is one of the main obstacles to healthy longevity in the world's aging society. A meta-analysis showed that the prevalence of chronic constipation is 10.1% when Rome IV criteria were used, and the incidence increases with age (1,2). The number of patients with chronic constipation is expected to increase in the future, and it has been reported that patients with chronic constipation have a worse prognosis (3,4). In daily clinical practice, constipation care is not always considered important, and we encounter elderly patients who are reluctant to go out into social environments for fear of abdominal pain, bloating, or diarrhea when laxatives are used. Stimulant laxatives are relatively common, but when used chronically, they have several side effects, including abdominal pain, diarrhea, and the development of tolerance (5). There is increasing clinical evidence that probiotics are effective for gastrointestinal disorders, although evidence is still insufficient to support the routine clinical use of probiotics with the exception of acute infectious diarrhea, antibiotic-associated diarrhea, *Clostridium difficile*–associated diarrhea, and necrotizing enterocolitis in preterm infants with fewer side effects (2,6). In addition, there is no sufficient evidence for the effects of probiotics on chronic constipation, especially in elderly individuals.

In recent years, reports using next-generation sequencing have shown that the amount of bifidobacteria is reduced in the stools of elderly individuals (7). The relationship between chronic constipation and gut microbiota has attracted attention. *Bifidobacterium longum* BB536, originally isolated from a healthy infant, is a clinically effective, well-established, multifunctional probiotic that has a long history of human use in alleviating gastrointestinal, immunological, and infectious diseases (8). *B. longum* BB536 has been found to be effective for abnormal bowel movements when given with dairy products (9). In comparison to standard yogurt fermented with starter cultures (*Streptococcus thermophilus* and *Lactobacillus bulgaricus*), supplementation of BB536 yogurt (fermented with starter cultures plus *B. longum* BB536) led to an increased abundance of Bifidobacterium and improved intestinal environments (such as lowered fecal levels of ammonia and increased levels of short chain and volatile acids) (9,10). The powdered form of this strain has been reported to be useful for elderly patients receiving enteral feeding (11). In this study, Kondo et al. reported that there was no intergroup difference in the overall frequency of defecation for either treatment, but subgroup analyses based on the baseline frequency of defecation revealed significant increases in bowel movements in patients with a low frequency of defecation and significant decreases in the bowel movements of patients with a high frequency after the intervention in the BB536 groups. Nevertheless, the efficacy of BB536 powder for chronic constipation in elderly patients undergoing outpatient care and the changes in the gut microbiota caused by probiotic administration have not been investigated in detail. Therefore, we aimed to perform a double-blind, randomized, controlled trial of this strain to verify its efficacy.

## METHODS

### Design overview

This study was performed with the approval of the ethical committee of Juntendo University and was based on the tenets of the Declaration of Helsinki. Informed consent was obtained from each participant. This study is registered at the University Hospital Medical Research Network, number UMIN000033031.

### Setting and participants

This study was conducted on outpatients attending the Department of Gastroenterology at the Juntendo Tokyo Koto Geriatric Medical Center. The inclusion criteria were as follows: (i) male and female patients aged 65 years or older at the time of consent, (ii) patients diagnosed with functional constipation or constipated irritable bowel syndrome according to the Rome IV diagnostic criteria, (iii) patients who were diagnosed with a Constipation Scoring System (CSS) score of 6 or higher, and (iv) patients who provided written informed consent for participation in this study. The exclusion criteria were as follows: (i) organic constipation due to colorectal cancer, colorectal or anal stenosis, a rectal mass or rectal overload, pseudo bowel obstruction, or a giant rectum; (ii) neurological disease due to a spinal cord lesion, cerebral infarction, Parkinson disease, or multiple sclerosis; (iii) diabetes mellitus, hypercalcemia, hypokalemia, hypomagnesemia, hypothyroidism, or uremia; (iv) use of opioids, anticholinergics, calcium channel blockers, anticonvulsants, psychotropic drugs, antispasmodics, histamine H1-receptor antagonists, or antiemetics; (v) amyloidosis, systemic scleroderma, or heavy metal poisoning; (vi) serious cerebrovascular disease, hepatic disease, renal disease, gastrointestinal disease, endocrine/metabolic disease, an infectious disease requiring notification, a history of cancer of the digestive system, currently receiving treatment/medication for the disease, a history of major surgery in the digestive system such as gastrectomy, gastrointestinal suturing, or intestinal resection, or digestive disorders such as infectious enteritis or inflammatory bowel syndrome; (vii) the regular use of medications that affect their bowel movements (antibiotics, bowel control, antidiarrheals, etc.), or certain health foods and supplements (lactic acid bacteria, bifidobacteria, oligosaccharides, dietary fiber, etc); (viii) significant abnormalities in blood pressure/blood tests, severe anemia, or allergies to drugs or foods; (ix) excessive smoking, regular use of alcohol, an irregular diet, an abnormal sleep cycle, or other lifestyle abnormalities; and (x) in addition to the above, the principal investigator may deem any patient to be ineligible.

### Randomization, intervention, and evaluations

After the assessment for eligibility, randomization was conducted using random permuted blocks of the participants after they were stratified by sex (male vs female) and CSS score (≥9 vs <9) to ensure a balanced allocation of the participants in the probiotic (BB536) and placebo groups. Sachets containing lyophilized powder of *B. longum* BB536 (5 × 10^10^ colony-forming unit or more, 2 g/package) or placebo were prepared as previously described (8). Each participant consumed 1 probiotic or placebo sachet daily for 4 weeks and was asked to participate in 4 weeks of postobservation. All of the members of the research team were unaware of the allocated sequence until the end of the study and when the database was locked.

The patients were assessed by 2 questionnaires: the CSS (12) (see Supplementary Table 1, http://links.lww.com/AJG/C706) and frequency scale for the symptoms of gastrointestinal reflux disease (FSSG; gastrointestinal reflux disease [GERD]) (13). The questionnaires were given to the patients at the outpatient visits before (baseline, week 0) and after (week 4) the initiation of the intervention and at the end of the period (week 8). The CSS questionnaire evaluated a total of 8 items, including (i) frequency of bowel movements, (ii) difficulty: painful evacuation effort, (iii) completeness: feeling incomplete evacuation, (iv) pain: abdominal pain, (v) time: minutes in lavatory per attempt, (vi) assistance: type of assistance (laxatives, enemas, or manual maneuvers), (vii) failure: unsuccessful attempts for evacuation every 24 hours, and (viii) history: duration of constipation (years). The scores of the 8 items were summarized as the total CSS score. The FSSG scale, which included a total of 12 items, was used to evaluate the symptoms of GERD. The changes in the patients' scores from baseline to week 4 and week 8 were also examined. The total CSS after intervention (week 4) was the primary end point, and the FSSG total score and the subscale scores of CSS and GERD as well as the changed scores from baseline were the secondary end points.

### Fecal DNA preparation, microbiota analysis, and microbiota function

Fecal samples were collected before and after the intervention. The fecal DNA preparation and microbiota analysis were performed as previously described (14). In brief, the fecal samples were collected using Techno Suruga stool collection kit brush type at week 0 and week 4. DNA was extracted from the fecal samples, and the purified DNA was suspended in 2,000 mL of Tris-EDTA buffer (pH 8.0). Polymerase chain reaction amplification and DNA sequencing of the V3–V4 region of the bacterial 16S rRNA gene were performed on an Illumina MiSeq instrument (Illumina, San Diego, CA) as previously described (15). After removing the sequences consistent with the data from the Genome Reference Consortium human build 38 (GRCh38) and the phiX reads from the raw Illumina paired-end reads, the sequences were analyzed using the QIIME2 software package (version 2017.10) (1). Potential chimeric sequences were removed using Divisive Amplicon Denoising Algorithm 2 (DADA2) (16), and 30 and 90 bases were trimmed from the 30 regions of the forward and reverse reads, respectively. The taxonomical classification was performed using the naive Bayes classifier that had been trained on the Greengenes 13.8 data set with a 99% sequence similarity threshold for full-length operational taxonomic units. A principal coordinate analysis based on the Bray‒Curtis distance was performed using Quantitative Insights Into Microbial Ecology 2 (QIIME2) software. Phylogenetic Investigation of Communities by Reconstruction of Unobserved States 2 (PICRUSt2) (17) was used to infer the gut microbial functional genes based on the microbiota composition of 16S rRNA gene sequences with default settings.

### Statistical analysis

Assuming that the treatment effect is expected to be equivalent to that of a previous study that investigated the efficacy of a traditional Japanese medicine in improving functional constipation (18), the group difference in the CSS mean of the pretreatment and posttreatment differences was set as −2.5, the SD of the measurements as 4, the correlation coefficient between the pretreatment and posttreatment measurements as 0.6, and the SD of the pretreatment and posttreatment differences as 3.58. Therefore, to detect a difference of −2.5 between the groups and to obtain 80% power by the 2-sample *t* test, 34 participants in each group (68 participants in total) were needed. After considering patient dropouts and other factors, we increased the number of participants by approximately 15%, making a total of 80 participants the target number of cases.

Once the data collection was completed, all data were fixed before the code-breaking. The primary end point was the difference in the changes from baseline in the CSS total score between the 2 groups at week 4. The changes from baseline were compared between the probiotic and placebo groups using the Wilcoxon rank-sum test. Moreover, the intragroup changes in the values between the baseline and after intervention were tested using the Wilcoxon signed-rank test. The statistical analysis was performed using SAS software version 9.4 (SAS Institute, Cary, NC) for the gastrointestinal data and R software ver. 3.6.0 for the gut microbiota data, with significance set at *P* < 0.05.

## RESULTS

**Figure 1.**
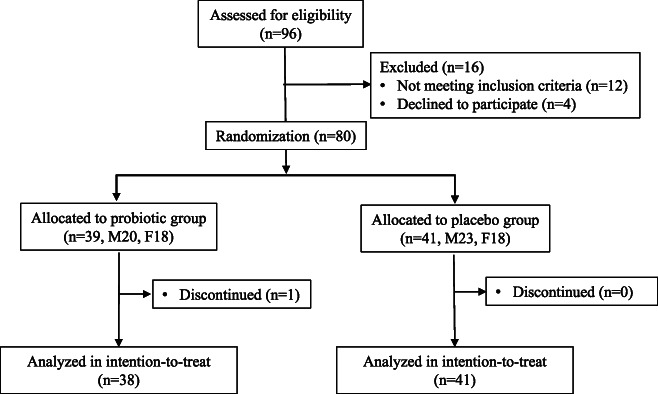
Trial profile.

Ninety-six patients were evaluated for eligibility, and 80 were randomly assigned (Figure 1). There were 80 patients (M/F: 36/44, mean age 77.9 years), including 39 patients in the probiotic group and 41 in the placebo group, and there were no differences in the patient backgrounds between the groups (Table 1). One patient in the probiotic group dropped out because of difficulty in going to the hospital, which left 38 patients in the probiotic group and 41 patients in the placebo group who were evaluated. In both groups, the dose rate was almost 80% or more.

**Table 1.**
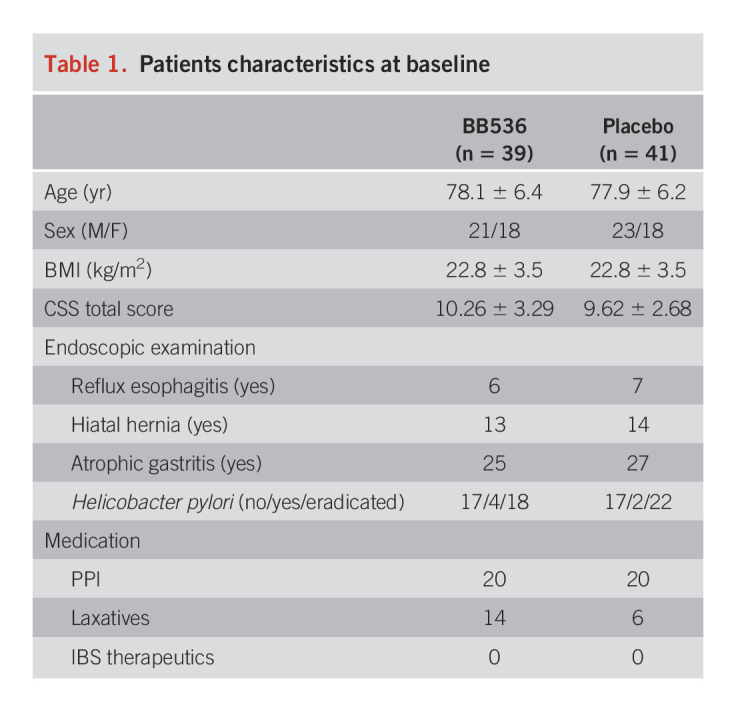
Patients characteristics at baseline

### Bowel movements

**Figure 2.**
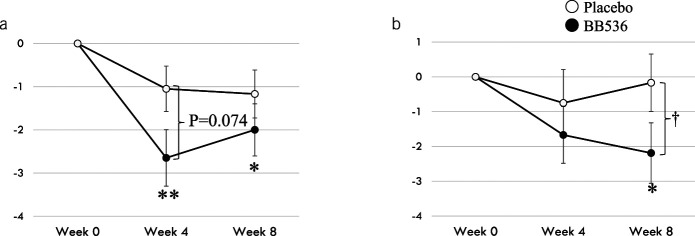
Effect of *Bifidobacterium longum* BB536 administration on the clinical symptoms in elderly individuals with chronic constipation. (**a**) Changes in the Constipation Scoring System (CSS) scores from baseline. (**b**) Changes in the frequency scale for the symptoms of gastrointestinal reflux disease scores (gastrointestinal reflux disease [GERD]) from baseline. *P < 0.05, **P < 0.01, significant difference from baseline (Wilcoxon signed-rank test); †*P* < 0.05, significant difference compared to the placebo group (Wilcoxon rank-sum test).

Table 2 shows the results of the CSS. Significant improvement was observed in the CSS score from baseline to weeks 4 (after the intervention, *P* < 0.01) and 8 (after the postobservation period, *P* < 0.05) in the probiotic group, but there were no significant changes in the placebo group; however, no intergroup difference was observed. There was a tendency for a difference in the changes in the CSS scores from baseline to after the intervention between the 2 groups (week 4, *P* = 0.074, Figure 2a). There were several items that tended to be improved after the treatment in the probiotic group, but these items were not improved in the placebo group; a significant intergroup difference was observed in failure of evacuation (unsuccessful attempts for evacuation per 24 hours) after the intervention (week 4). In addition, there were intergroup differences in the changes in the subscale items at week 4 from baseline, such as the stool frequency (*P* = 0.008) and failure of evacuation (*P* = 0.051).

**Table 2.**
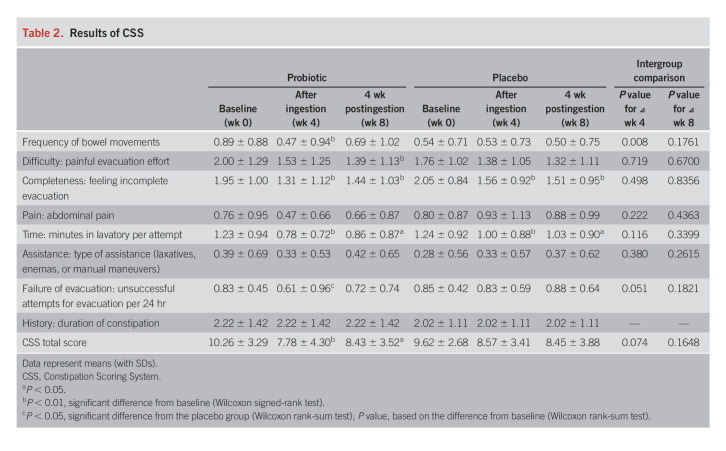
Results of CSS

On the FSSG scale (Table 3), no improvement was observed in the placebo group, but improvements were observed in the heartburn and the treatment getting stuck while swallowing subscales in the probiotic group after the intervention (*P* < 0.05). In the intergroup comparison of the variable values, the heartburn (*P* = 0.089) and feeling sick after meals (*P* = 0.075) subscales tended to improve after 4 weeks of intake compared with the placebo group. Interestingly, although there was no significant difference after the intervention (week 4), improvement of the symptom scores was observed at the postintervention assessment (week 8) for stomach bloating (*P* = 0.032), unusual sensation in throat (*P* = 0.039), and getting stuck while swallowing (*P* = 0.057) in the probiotic group compared with the placebo group. There was no significant change in the total FSSG score after the intervention (week 4) in the placebo group, but a significant improvement was observed in the probiotic group (*P* < 0.05), with a tendency toward an intergroup difference (*P* = 0.097) at the postintervention assessment (week 8) (Figure 2b).

**Table 3.**
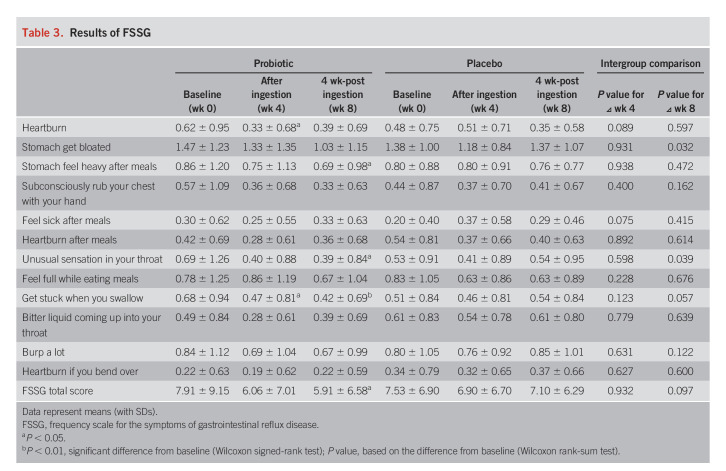
Results of FSSG

### Gut microbiota and microbiota function

**Figure 3.**
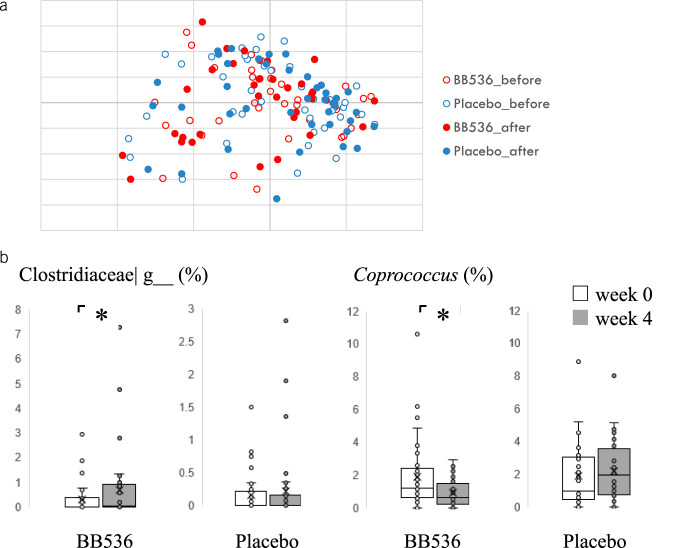
Effect of *Bifidobacterium longum* BB536 administration on the gut microbiota composition. (**a**) Bray–Curtis distance principal coordinate analysis (PCoA). (**b**) Differences in the genera before and after administration in each group. *P < 0.05 (Wilcoxon signed-rank test).

The Bray–Curtis principal coordinate analysis based on the genus level composition showed no significant difference between the groups and before and after the treatment intake (Figure 3a). We found that the relative abundance of *Clostridiaceae*|g increased (*P* = 0.042) and that of *Coprococcus* decreased (*P* = 0.045) in the BB536 group at week 4 compared with baseline (Figure 3b), although these differences were not significant with false discovery rate (FDR) correction (see Supplementary Table 2, http://links.lww.com/AJG/C707). We then inferred the gut microbial functional genes based on the microbiota composition by PICRUSt2. We found 2 and 14 differential pathways between the groups at 0 and 4 weeks, respectively (see Supplementary Figure 1 and Table 3, http://links.lww.com/AJG/C704, http://links.lww.com/AJG/C708). We also observed 1 and 3 intragroup differences between 0 and 4 weeks in the placebo and probiotics groups, respectively (see Supplementary Appendix Figure 2 and Table 4, http://links.lww.com/AJG/C705, http://links.lww.com/AJG/C709). However, these differences in the pathway were not significant with FDR correction.

### Safety

No deaths occurred in the trial. One case of diarrhea was observed during treatment intake in both the BB536 and placebo groups. There were no differences in the incidence of serious, total adverse events, or withdrawals due to adverse events between the BB536 and placebo groups. There were no adverse events secondary to the administration of the treatment.

## DISCUSSION

In this study, we investigated the effects of BB536 on constipation and abdominal symptoms in elderly patients with chronic constipation using the CSS and FSSG questionnaires. This is the first randomized controlled trial evaluating BB536 for chronic constipation in elderly outpatients. Although there was no intergroup difference in the total CSS (the primary outcome), we observed an improvement in the patients' bowel movements and upper abdominal symptoms after 4 weeks of BB536 intake compared with the placebo group. Interestingly, improvements in the symptom scores were observed even 4 weeks after treatment intake. To the best of our knowledge, this is the first study to see a prolonged effect of probiotics on constipation and upper abdominal symptoms in elderly patients with chronic constipation. This prolonged effect may be caused by the improvement of the intestinal environment owing to the 4-week probiotic intake.

Previous studies have reported the efficacy of probiotics for chronic constipation (19,20). A systematic review of chronic constipation in the elderly was previously reported (21). In a systematic review, *B. longum, B. lactis, Lactobacillus, Bifidobacterium,* and *Streptococcus* were administered in combination with a fermented oat drink or as a powder (11,22,23). However, the patients in these clinical trials were not diagnosed with chronic constipation, and these studies included patients who had diarrhea (11). In addition, the subjects were hospitalized patients or patients who received tube feeding. Therefore, there are many biases in these studies.

*Bifidobacterium* has been reported to promote intestinal peristalsis through the production of short-chain fatty acids such as acetic acid (24–26). These short-chain fatty acids affect the gut microbiota and improve bowel movement (15). Although we did not measure the change in intestinal metabolites, previous studies have demonstrated effectiveness in the improvement of intestinal environments, such as increased levels of acetic acid and butyric acid, by the intake of BB536 yogurt (11). *Bifidobacterium* is well known for acetic acid production, and *B. longum* BB536 has been demonstrated to promote the level of butyric acid mediated by crosstalk with other bacteria in the gut microbiota (27–29). Therefore, *B. longum* BB536 may contribute to an improvement in defecation abnormalities, such as an improvement in the number of defecations and defecation difficulties, in these patients.

In recent years, the overlap of functional gastrointestinal diseases has been reported, such as dyspepsia and constipation (30,31). In functional gastroenteropathy, the effect of inflammation of the duodenum has been reported (32), and microinflammation of the mucosa of the gastrointestinal tract is believed to have an influence. *Bifidobacterium* has been reported to improve intestinal barrier function and suppress inflammation (27). *B. longum* BB536 was reported to be useful for allergic diseases such as hay fever and is believed to regulate the immune balance through the intestinal immune system and to suppress inflammation (33). Therefore, it was believed that the suppression of inflammation improves these patients' upper abdominal symptoms as well.

In this study, we did not find a marked change in the gut microbiota composition after treatment intake. The relative abundance of *Clostridiaceae*| g increased and that of *Coprococcus* decreased in the BB536 group at week 4 compared with baseline. We found that the relative abundance of *Coprococcus* tended to have a positive correlation with the CSS score at week 4 in the BB536 group; however, there was no obvious difference in the changes in the CSS scores from baseline between the *Coprococcus*-increased group and *Coprococcus*-decreased group, and there was no correlation of *Coprococcus* with the CSS score at 0 weeks (data not shown). Our data suggest that the relative abundance of this genus did not seem to affect the CSS score. This suggests that changes in metabolic and physiological pathways mediated by bacterial components and metabolites such as acetic acid of BB536, rather than changes in the composition of the microbiota, may affect defecation. Similar observations were made by Kaczmarczyk et al. (34), who found that probiotic intervention modified the biochemical and physiological parameters, regardless of changes in microbiota composition and metabolic function over time. McNulty et al. (35) observed a change in the metabolic functions of the microbiota under the influence of probiotics, despite no change in its composition. In the present study, PICRUSt2 analysis indicated more MetaCyc differential pathways between the groups at postintervention than at baseline. Four weeks of BB536 intervention also seems to result in more differential MetaCyc pathways compared with the placebo group. However, these differences in the pathway were not significant with FDR correction, and the association with clinical observation awaits future investigation. On the other hand, because we could not observe a significant improvement in the total CSS, the relationship between gut microbiota changes and the clinical efficacy that was observed in the present study awaits further investigation in studies with a larger sample size.

In this study, we reported that BB536 improved defecation and some upper abdominal symptoms in elderly patients with chronic constipation. It was observed that some of the improved symptoms were maintained even 4 weeks after stopping the probiotics. This probiotic therapy had very few adverse effects. These results suggest the safety and usefulness of taking *B. longum* BB536 for chronic constipation in elderly individuals. However, several limitations exist for this study. First, the study included a relatively small number of individuals. Second, we did not assess lifestyle habits such as diet, alcohol consumption, and exercise habits during the intervention. Third, we did not follow-up with patients for a long period. This study had several strengths, including being a randomized, double-blind, placebo-controlled parallel intervention study, which included a detailed evaluation of the constipation symptoms and the upper abdominal symptoms. This study also included an evaluation of the fecal microbiota. In the future, it is necessary to examine a large number of cases at multiple institutions and to analyze metabolites such as organic acids in the intestinal tract.

## CONFLICTS OF INTEREST

**Guarantors of the article:** Tsutomu Takeda, MD, PhD, Jin-Zhong Xiao, PhD, and Toshifumi Ohkusa, MD, PhD, AGAF.

**Specific author contributions:** T.T., D.A., and T. Ohkusa: designed the study. T.T. and D.A.: recruited the patients, collected the data, and performed the analyses. T. Osada: allocated the cases. S.K., N.K., T. Odamaki, and J.X.: analyzed the fecal microbiota. S.N., N.Y., and Y.N.: performed the statistical analyses. S.N., N.Y., and Y.N.: performed the statistical analyses. T.T.: prepared the initial manuscript; D.A. and T. Ohkusa: revised the manuscript; and A.N. and N.S.: supervised the study. All authors read and approved the final manuscript.

**Financial support:** Funding was provided by the Department of Microbiota Research, Juntendo University Graduate School of Medicine.

**Potential competing interests:** T.T., D.A., S.K., N.K., T.O., J.X., T.O., and N.S. are members of the Department of Microbiota Research, Juntendo University Graduate School of Medicine.

**Clinical trial registration:** This study protocol was registered on UMIN-CTR (https://www.umin.ac.jp/icdr/index.html), and the trial identification number is UMIN 000033031.

**Institutional review board statement:** This study was conducted in compliance with the Declaration of Helsinki (Fortaleza, Brazil 2013) and the Ethical Guidelines for Medical Research Involving Human Subjects (2014) of the Ministry of Education, Culture, Sports, Science and Technology and the Ministry of Health, Labor and Welfare. This study protocol was approved by the Ethics Committee of Juntendo Tokyo Koto Geriatric Medical Center Institutional Review Board (No. 98-3).

**Informed consent statement:** Informed consent was obtained from all subjects involved in the study. Written informed consent was obtained from the patients to publish this paper.

**Data availability statement:** The data sets and materials in this study are available from the corresponding author.

**Study Highlights**

**WHAT IS KNOWN**

✓ Chronic constipation increases with age.
✓ Conventional laxatives have many side effects, and when used continuously, they become resistant, and their effects are diminished.
✓ Previous studies have reported the efficacy of probiotics for chronic constipation and have fewer side effects.
✓ Few reports exist regarding the therapeutic effects of probiotics on chronic constipation in elderly individuals.

**WHAT IS NEW HERE**

✓ This study is the first randomized controlled trial evaluating *Bifidobacterium longum* for chronic constipation in elderly outpatients.
✓ Significant improvement was observed in the changed values of frequency of bowel movements after 4 weeks of *B. longum* intake compared with the placebo group.
✓ Marked changes in the gut microbiota composition were not found after the treatment intake, suggesting that bacterial components and metabolites of *B. longum*, rather than the changes in microbiota, may affect defecation.

## Supplementary Material

SUPPLEMENTARY MATERIAL
